# Cardiac auscultation via simulation: a survey of the approach of UK medical schools

**DOI:** 10.1186/s13104-015-1419-y

**Published:** 2015-09-10

**Authors:** Samantha Jayne Owen, Kenneth Wong

**Affiliations:** Hull York Medical School, John Hughlings Jackson Building, Heslington, York, YO10 5DD UK; Department of Academic Cardiology, Hull York Medical School, Castle Hill Hospital, Castle Rd, Cottingham, Hull, HU16 5JQ UK

**Keywords:** Simulation, Medical education, Auscultation, Curriculum

## Abstract

**Background:**

A decline in clinical skills of medical students and junior doctors is well documented. We aim to determine how the 32 UK medical schools utilise simulated heart sounds to develop medical students’ cardiac auscultation skills.

**Methods:**

Representatives of all 32 UK medical schools were contacted with a survey questionnaire. Data analysis was carried out using SPSS. Continuous variables e.g. teaching group size were described using median and interquartile range (IQR).

**Results:**

27 Medical schools use a form of simulated heart sounds as a teaching method (2 representatives were unsure, 3 did not respond). This teaching is mandatory in 17 schools. Simulation-based teaching tends to be offered during 3rd year of medical training [median = 3rd year, interquartile range (IQR) 2–3]. Seven medical schools offered simulation teaching more than once. The median number of students in each session was 7.5 [IQR = 5.5–9.5]. One medical school reported that they were unsure how best to implement the heart sound simulation into the medical undergraduate curriculum.

**Conclusion:**

The results of our survey of all the UK medical schools suggest that heart sound simulation are used mainly as an introduction to heart murmurs rather than a tool for repetitive practice, complementing clinical experience. Most medical schools do not measure the impact of such teaching on clinical examination.

## Background

Clinical skills are important and are recognised as being fundamental to the practice of medicine. A decline in the clinical skills of medical students and junior doctors is well documented [1–3]. Cardiac auscultation skills have been noted to be particularly deficient [4–6]. Recognising auscultatory sounds forms a vital part of the cardiovascular examination yet accuracy for identifying specific diagnoses has been shown to be low amongst medical students and trainees [5]. There is a tendency to interpret healthy heart sounds as cardiac murmurs and abnormalities are frequently missed. Poor cardiac auscultation skills may not only miss important pathology but could result in over-diagnosing a patient, leading to financial implications in terms of unnecessary referral for echocardiography [3].

Audio recordings of cardiac murmurs have served as educational tools for several years and have the advantage of being easily reproduced outside the ward. More sophisticated heart sound simulators such as ‘Harvey’ offer a more realistic patient encounter. Simulated heart sounds have shown promise in research as an effective educational strategy for teaching cardiac auscultation skills, which may justify their widespread use in medical curricula [6–9]. Given that simulation based cardiac auscultation training has been repeatedly shown to improve cardiac auscultation skills, why are the skills of medical students and trainee doctors declining? Are all UK medical schools offering simulation as part of their undergraduate medical curriculum? If so, are they using it effectively? There has been no study to determine how UK medical schools utilise simulated heart sounds for the teaching of auscultation skills throughout the undergraduate medical curriculum.

We aim to determine how the 32 UK medical schools utilise simulated heart sounds to develop medical students’ cardiac auscultation skills.

## Methods

The research was carried out in compliance with the Helsinki Declaration and ethical approval was granted from HYMS ethical committee. Clinical skill leads or equivalent from the 32 UK medical schools were contacted directly via email and telephone. Clinical skills leads referred to members of university staff that were responsible for designing the cardiovascular component of the medical school curriculum and deciding how clinical examination of the cardiovascular system would be taught in their respective schools. They were informed that we were doing a research study to try to find out how UK medical schools utilise simulated heart sounds to develop medical students’ cardiac auscultation skills.

## Results

**Fig. 1:**
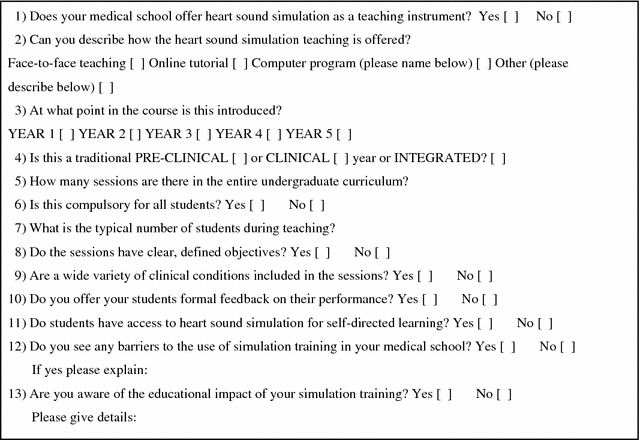
Simulation-based cardiac auscultation skills survey

Twenty nine out of the 32 UK medical schools returned the cardiac simulation questionnaire (Fig. 1). Two of these 29 respondents indicated they did not know enough about how their cardiac simulation training was delivered to their students to fill in the survey. Data for 27 medical schools was subsequently analysed.

### Utilisation of simulated heart sounds

All 27 medical schools use a form of simulated heart sounds as a teaching method. For students at 17 of these medical schools, this teaching is mandatory. Eight medical schools said this teaching was optional and two medical schools did not answer this question.

### Method of delivery

Simulation based teaching tends to be offered during 3rd year of medical training [median = 3rd year, interquartile range (IQR) 2–3]. 66 % of the medical schools introduce heart sound simulation training within an integrated curriculum, whilst 4 introduced this form of training during the clinical course (12.5 %). Seven did not answer this question whether simulation training was introduced in a traditionally preclinical, clinical or integrated year.

The median number of students in each session was 7.5 [IQR = 5.5–9.5]. Only 9 responses provided this numerical data. The teaching took the form of face-to-face teaching rather than online tutorial or computer programmes and 6 medical schools use ‘Harvey’. Students have access to simulated heart sounds for self-directed learning in 11 medical schools. One medical school reported that they were unsure how best to implement the heart sound simulation into the medical undergraduate curriculum.

The majority of medical schools would offer simulation based teaching as an introduction to the topic of heart murmurs only, with a maximum of 4 h of teaching in the entire undergraduate curriculum. Five medical schools offered simulation teaching more than once, with two medical schools offering it three times during the undergraduate curriculum. However, median number of times offered during the course was 1, [IQR, 1–1].

Only 1 medical school said that their sessions had clear, defined objectives, however all medical school thought that their simulation training covered a wide range of clinical conditions. Two medical schools said they gave their students formal feedback after the sessions. Not one medical school was aware of the education impact of simulation training although three medical schools (9.4 %) are currently evaluating the educational impact of this form of training. No medical school anticipated barriers to the implementation of simulations based training.

## Discussion

Twenty seven UK medical schools use simulated heart sounds as a teaching method and it tends to be offered during the 3rd year of medical training. The teaching is usually face-to-face and hands-on within a small group. Eleven medical schools allow their students access to simulated heart sounds for self-directed learning, however the value of this in the absence of formal teaching in unknown.

There are a number of studies that have showed that increasing the opportunity for repetitive and deliberate practice through direct contact with the simulator appears to increase the effectiveness of cardiac auscultation skills acquisition [7, 8]. A more recent presentation given by the authors at the ASME Annual Scientific Meeting details how, by undergoing a selected student component which utilised simulation as a way of teaching heart sounds, students at Hull York Medical School were significantly more able to accurately diagnosis heart murmurs correctly [10]. Our study, involving a survey of all the UK medical schools, indicates that the majority are only offering simulation-based teaching once during the undergraduate curriculum, usually as an introduction to simulation, upon which students are expected to develop as self-directed learning. The majority of UK medical schools use simulation-based auscultation teaching as part of an integrated curriculum usually in the third year. This is presumably because this is the first year of full time clinical attachments for the majority of students where these skills should, in theory, prove the most useful.

Important features of simulation-based medical education reported in the literature include, the use of clearly stated goals, using simulators that capture a wide variety of clinical conditions with a range of difficulty level, use of simulators in a controlled environment where learners can make, detect and correct errors, and importantly, providing students with feedback on their performance [8]. Only 1 medical school said that their sessions had clear, defined objectives, however all medical school thought that their simulation training covered a wide range of clinical conditions. Two medical schools said they gave their students formal feedback after the session. This implies that there could be some improvements made in the delivery of simulation based training in order to achieve a greater educational impact. The majority of medical schools would offer simulation based teaching as an introduction to the topic of heart murmurs only, with a maximum of 4 h of teaching in the entire undergraduate curriculum. An increase in the amount of time that is engaged in learning simulation-based cardiac physical examination has been shown to lead to better learning outcomes [6]. Medical schools could be encouraged to increase the amount of teaching of this kind if possible, and give detailed and prompt feedback to their students, so as to increase the educational impact of this style of teaching.

Two medical schools were reviewing the impact of auscultation teaching on medical school examination results, however the majority of medical schools did not monitor the outcome of simulation based training in terms of an improvement in their students’ cardiac auscultation skills or success in medical school examinations.

## Limitations

Clinical skills leads, people heavily involved in the teaching of clinical skills, were sought to take part in the survey. It was likely that these representatives knew accurately how cardiac auscultation skills with the medical school were taught and how simulation is utilised. However, as medical curricula are very diverse, it is possible that the representatives asked may have very different roles, backgrounds and levels of awareness of the curriculum.

The replies to the survey questions were based solely on the impression of the respondent at each medical school which could be perceived as being subjective.

## Conclusion

The results of our survey of all the UK medical schools suggest that heart sound simulation technology is not utilised to its maximum benefit. In UK medical schools heart sound simulators are used mainly as an introduction to heart murmurs rather than a tool for repetitive practice to complement clinical experience. Most medical schools do not measure the impact of such teaching on clinical examination performance to see if teaching is effective or how it compares to learning from other methods such as auscultating more patients on the bedside.

The authors appreciate that the value of the study is greatest to those with closely related research interests. Nonetheless, they feel it is helpful to have a current knowledge of the way medical schools are utilising simulated heart sounds in their teaching, given that the skill of cardiac auscultation is in decline and the reasons for this decline warrants further study.
